# Mutant Versions of the *S. cerevisiae* Transcription Elongation Factor Spt16 Define Regions of Spt16 That Functionally Interact with Histone H3

**DOI:** 10.1371/journal.pone.0020847

**Published:** 2011-06-06

**Authors:** Catherine N. Myers, Gary B. Berner, Joseph H. Holthoff, Kirby Martinez-Fonts, Jennifer A. Harper, Sarah Alford, Megan N. Taylor, Andrea A. Duina

**Affiliations:** Biology Department, Hendrix College, Conway, Arkansas, United States of America; Duke University, United States of America

## Abstract

In eukaryotic cells, the highly conserved FACT (FAcilitates Chromatin Transcription) complex plays important roles in several chromatin-based processes including transcription initiation and elongation. During transcription elongation, the FACT complex interacts directly with nucleosomes to facilitate histone removal upon RNA polymerase II (Pol II) passage and assists in the reconstitution of nucleosomes following Pol II passage. Although the contribution of the FACT complex to the process of transcription elongation has been well established, the mechanisms that govern interactions between FACT and chromatin still remain to be fully elucidated. Using the budding yeast *Saccharomyces cerevisiae* as a model system, we provide evidence that the middle domain of the FACT subunit Spt16 – the Spt16-M domain – is involved in functional interactions with histone H3. Our results show that the Spt16-M domain plays a role in the prevention of cryptic intragenic transcription during transcription elongation and also suggest that the Spt16-M domain has a function in regulating dissociation of Spt16 from chromatin at the end of the transcription process. We also provide evidence for a role for the extreme carboxy terminus of Spt16 in functional interactions with histone H3. Taken together, our studies point to previously undescribed roles for the Spt16 M-domain and extreme carboxy terminus in regulating interactions between Spt16 and chromatin during the process of transcription elongation.

## Introduction

In eukaryotic cells, genomic DNA associates with several proteins to form a protein-DNA structure referred to as chromatin. The fundamental unit of chromatin is the nucleosome, a structure composed of 147 base pairs of DNA associated with a protein complex known as the histone octamer [1]. In addition to assisting in the compaction of DNA within cell nuclei, nucleosomes are also crucial participants in regulating a variety of processes that occur in the context of chromatin, including the process of gene transcription [2]. In general, the presence of nucleosomes has repressive effects on productive transcription since nucleosomes often compete for access to the DNA with factors that promote transcription, such as transcription activators and RNA polymerase II (Pol II), the multi-protein complex responsible for synthesizing RNA from protein-encoding genes [3], [4].

An area of intense research in recent years has focused on elucidating the mechanisms that allow for productive Pol II passage over transcribed units despite the presence of nucleosomes across these regions. These studies have identified a large number of factors possessing a variety of biochemical activities that have been implicated in the process of transcription elongation in the context of chromatin [5]. One of the critical players in this process is FACT, a highly conserved and abundant heterodimeric complex. The identification of the components of this complex and initial insights into its function came through genetic and biochemical studies in the *Saccharomyces cerevisiae* model system [6], [7], [8], [9]. In subsequent landmark experiments using human cell extracts, the human FACT complex was isolated as an activity required for productive transcription elongation on nucleosomal templates *in vitro* [10]. More recent studies have led to a model for FACT function during transcription elongation in which the complex directly associates with nucleosomes to form a FACT-histones-DNA structure referred to as the reorganized nucleosome, which in turn facilitates removal of H2A-H2B dimers upon Pol II passage, thereby promoting Pol II elongation [11], [12], [13]. The FACT complex is then thought to assist in the reassembly of the histones into nucleosomes following Pol II passage [14], [15]. In recent experiments, it has been shown that the histones H3 and H4 that are reassembled into nucleosomes in the wake of Pol II passage correspond to the ones that were originally displaced during the elongation process, but only when FACT is functional [16]. Although the FACT complex was originally named after the biochemical function that allowed its purification (FAcilitates Chromatin Transcription) [10], some have proposed the same acronym be used to stand for FAcilitates Chromatin Transactions to more accurately reflect the varied chromatin-based processes that depend on FACT, including transcription initiation and DNA replication [12], [17].

The yeast FACT complex (yFACT) is composed of two subunits essential for viability, Spt16 and Pob3, which can interact with nucleosomes through the assistance of the HMG box-containing protein Nhp6 [18], [19], [20], [21]. X-ray crystallographic and structure-function studies of Pob3 and Spt16 have provided significant insights into the physical and functional organization of yFACT. Limited proteolysis experiments have shown that Pob3 contains three structurally distinct domains: an N-terminal domain referred to as Pob3-NTD/D, a central domain referred to as Pob3-M and an acidic C-terminal domain referred to as Pob3-C [22], [23]. Based on studies on human FACT, the Pob3-NTD/D domain is thought to be involved in dimerization with Spt16 [24]. The crystal structure of the Pob3-M domain revealed the presence of a double pleckstrin homology (PH) motif, which, in the context of Pob3-M, has been implicated in directing interactions with RPA, an essential protein involved in several DNA-based processes including DNA replication and repair [22].

Additional studies revealed the presence of four structural domains within the Spt16 subunit of yFACT – these domains are referred to, from the N-terminus to the C-terminus direction, as Spt16-NTD, Spt16-D, Spt16-M and Spt16-C [22], [23]. Several recent studies have shed light onto important features of the Spt16-NTD domain. Analyses of the crystal structures of the *S. cerevisiae* and *S. pombe* Spt16-NTDs show the presence of two potential peptide-binding modules – one within the N-terminal lobe of the Spt16-NTD and the other, structurally similar to bacterial aminopeptidases, in the more C-terminal lobe of the Spt16-NTD [25], [26]. In *S. cerevisiae*, the Spt16-NTD domain, in conjunction with the Pob3-M domain, is involved in functional interactions with the C-terminal extension of histone H2A [26], a region thought to be important for stabilizing interactions between H2A-H2B dimers and H3-H4 tetramers in the nucleosomal context [27], [28]. In *S. pombe*, the Spt16-NTD domain has been shown to interact directly with both the globular domains and N-terminal tails of histone H3 and H4 [25], implicating the Spt16-NTD in histone H3-H4 chaperoning onto DNA and/or in the formation of reorganized nucleosomes through H3-H4 interactions. However, given genetic data indicating that the Spt16-NTD is largely dispensable for Spt16 functions *in vivo* [23], other regions of Spt16 are likely to have critical roles in functional and physical interactions with histones. This is supported by evidence that certain truncations of the C-terminus of Spt16 are lethal in yeast [29] and by biochemical studies showing a requirement for the carboxy terminus of human Spt16 in FACT activity *in vitro* [15].

The role of the Spt16-M domain within yFACT is still under investigation. Mutations in residues within the Spt16-M domain have been shown to result in defects in cell integrity [30] and in phenotypes indicative of transcription initiation and elongation defects as well as defects in DNA replication [20]. However, the molecular functions for the Spt16-M domain and the relevant functional and physical interactions that occur between this domain and other proteins remain largely unexplored. In this study, we present evidence indicating that the Spt16-M domain is involved in functional interactions with histone H3. Our results indicate that the integrity of the Spt16-M domain is required to prevent cryptic intragenic transcription during the process of transcription elongation. We also provide evidence that, at least in certain contexts, the Spt16-M domain has roles in controlling Spt16 departure from chromatin at 3′ ends of transcribed genes. The extreme C-terminus of Spt16 is also shown to functionally interact with histone H3.

## Results

### Isolation of Spt16 mutants that genetically interact with histone H3

In previous work using *S. cerevisiae* as a model system, we described the isolation and characterization of a histone H3 mutant, H3-L61W, that confers several mutant phenotypes when expressed as the sole source of histone H3 in cells, including a cold sensitive (Cs^−^) phenotype and sensitivities to formamide, hydroxyurea and caffeine [31]. At the molecular level, H3-L61W was found to cause dramatic transcription-dependent alterations in the distribution of yFACT across transcribed genes, resulting in lower levels of the complex at 5′ ends of genes and in a marked increase in yFACT occupancy at the 3′ ends of genes [32], [33] (this phenotype will be referred to as the “3′-accumulation phenotype” throughout this report). Based on these and other data, we have proposed a model in which the H3-L61W mutation impedes dissociation of yFACT at the end of the transcription process – perhaps by preventing a yFACT-dissociation signal from reaching yFACT – thereby resulting in accumulation of yFACT at the 3′ ends of transcribed genes [32], [33]. We also showed that the H3-L61W mutation causes the generation of a cryptic transcript from within the *FLO8* gene [32], a phenotype associated with defective nucleosome reassembly in the wake of Pol II passage during transcription elongation [34], [35], [36], [37]. In the same study, we found that two independent mutations in Spt16, Spt16-E790K and Spt16-E857Q, partially suppress H3-L61W defects: both mutations showed suppression of the 3′-accumulation and *FLO8* cryptic transcription phenotypes, although the Spt16-E790K mutation only suppressed the latter phenotype at the low temperature of 14°C and not at the permissive temperature of 30°C [32]. The fact that the E790 and the E857 residues are located within the Spt16-M domain pointed us to the possibility that this domain might play important roles in interactions with histone H3.

**Figure 1:**
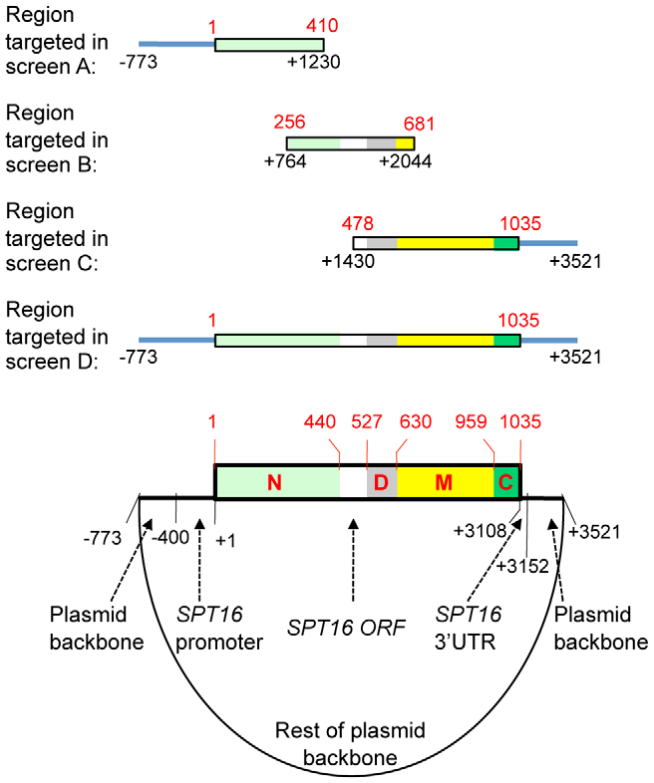
Illustration of the regions targeted for PCR-mediated mutagenesis in this study. The bottom diagram shows a cartoon representation of the *SPT16-*carrying plasmid pAO01, with the relevant regions indicated, that was used as template for PCR reactions in four independent screens (screens A, B, C and D). The numbering in black type refers to nucleotide positions across the region targeted for PCR mutagenesis and the numbering in red type refers to amino acid positions of the encoded Spt16 protein. The Spt16-NTD, Spt16-D, Spt16-M and Spt16-C domains defined in previous studies [22] are represented by the N, D, M and C letters and are also indicated by the light green, gray, yellow and dark green regions, respectively. The lines above the plasmid diagram show the regions of the plasmid, with the nucleotide and corresponding Spt16 amino acid positions and domains indicated, that were amplified by PCR in the four screens to give rise to different pools of randomly mutagenized fragments. See Materials and Methods section for further details pertaining to these experiments.

As a way to assess the potential importance of the Spt16-M domain in functional interactions with histone H3, we carried out unbiased screens to identify novel mutations across the entire length of the *SPT16* gene that suppress the Cs^−^ phenotype of H3-L61W cells. For these experiments, we PCR-amplified either different fragments (screens A, B and C) or the entire length (screen D) of the *SPT16* gene using the high-fidelity polymerase *Pfu* under standard PCR conditions (see Figure 1 and Material and Methods). These amplification conditions were found to produce an adequate yield of the desired mutations while minimizing secondary mutations. An H3-L61W strain carrying a deletion of the genomic *SPT16* locus and expressing wild-type Spt16 from a plasmid was then co-transformed with the PCR products and a linearized vector that had extensive homology with the PCR products. Following gap-repair and loss of the plasmid carrying wild-type *SPT16*, we then screened for the ability of the mutagenized *SPT16* genes to suppress the Cs^−^ phenotype caused by H3-L61W. Suppressing plasmids were then recovered from the yeast cells and the entire region under examination (from -773 to +3521, see Figure 1) was sequenced, regardless of which screen they originated from (see Materials and Methods).

Whereas a large proportion of the suppressing *spt16* mutants we isolated were found to have multiple mutations, fifteen mutants harbored either a single nucleotide change or two nucleotide changes (with one being a silent mutation within the *SPT16* coding region) across the region from −773 to +3521 and are the focus of the studies presented here. We were surprised to find that in several cases the mutations we identified in these mutants were located outside of the regions that were targeted by the PCR-mediated mutagenesis (see Table 1). Interestingly, we found that the mutations located outside the targeted regions were all transition mutations (6 out of 6 including silent mutations, see Table 1) and those located within the targeted regions were mostly transversion mutations (9 out of 12 including the silent mutations, see Table 1). These observations indicated to us that two modes of mutagenesis were in fact operating in our screens, one that tended to cause transversion mutations and the other that caused transition mutations. Since previous studies have reported that *Pfu* has a high propensity towards transversion mutations [38], [39], in particular GC to TA mutations [39], we attribute most of the mutations located within the targeted regions to *Pfu*-induced mutagenesis. On the other hand, we believe that the mutations found in regions located outside the targeted regions were likely due to exposure to UVB light during the manipulation of the PCR products and linearized plasmids since UVB-induced mutagenesis is associated with a high frequency of transition mutations [40]. These findings underscore the importance of sequencing the entire length of genes in experiments in which only small portions of the genes have been targeted by PCR-mediated mutagenesis.

**Table 1:** Summary of notable aspects pertaining to the four screens carried out in this study.

Screen	Spt16 amino acid residues included in targeted region	Estimated number of recombinant plasmids screened^1^	Spt16 mutants harboring single amino acid substitutions that suppress H3-L61W Cs^−^ phenotype^2^	Corresponding nucleotide change	Location of nucleotide change with respect to the targeted region	Type of mutation
A	1–410	1603	Spt16-R875K	G2624A	Outside	Transition
B	256–681	800	Spt16-P599Q	C1796A	Inside	Transversion
			Spt16-E735G^3^	A2204G	Outside	Transition
			Spt16-E735K	G2203A	Outside	Transition
			Spt16-G836S	G2506A	Outside	Transition
			Spt16-P838S	C2512T	Outside	Transition
C	478–1035	1141	Spt16-R712M	G2135T	Inside	Transversion
			Spt16-E790D	G2370T	Inside	Transversion
			Spt16-Q835K	C2503A	Inside	Transversion
			Spt16-P838T	C2512A	Inside	Transversion
			Spt16-Q854K	C2560A	Inside	Transversion
			Spt16-E989Stop	G2965T	Inside	Transversion
			Spt16-E1004Stop^4^	G3010T	Inside	Transversion
D	1–1035	2204	Spt16-K752E	A2254G	Inside	Transition
			Spt16-D787N^5^	G2359A	Inside	Transition

1 See Materials and Methods for the method used to estimate the number of recombinants screened in each experiment.

2 The mutants listed contain no additional mutations throughout the entire region examined (*i.e.* from –773 to +3521), except for silent mutations as indicated.

3 This mutant also contains a silent mutation (G2175A).

4 This mutant also contains a silent mutation (C1638A).

5 This mutant also contains a silent mutation (T1488C).

**Figure 2:**
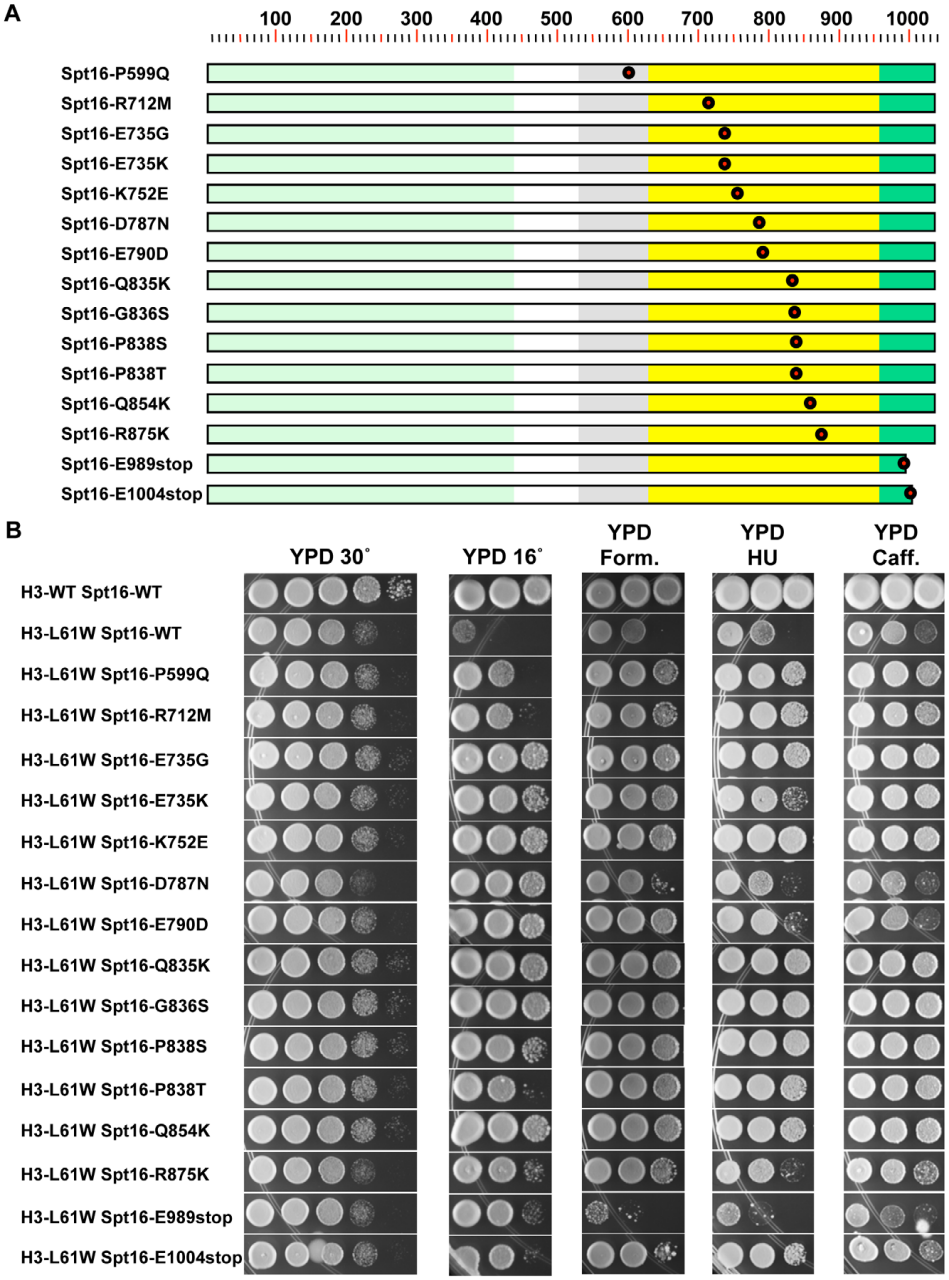
Initial characterization of newly isolated Spt16 mutants that suppress H3-L61W phenotypes. (A) Each mutant Spt16 protein is represented by a rectangle with the Spt16-NTD, Spt16-D, Spt16-M and Spt16-C domains indicated by the light green, gray, yellow and dark green regions, respectively. Approximate amino acid positions are provided by the numbers above the diagrams. The nature and location of the amino acid substitution or truncation in each case is included in the name assigned to the mutant proteins. The location of each mutated residue, or last residue for the truncation mutants, is also illustrated by a red circle along the length of the protein. Note that most of the amino acid substitutions are located within the Spt16-M domain. (B) Suppression profiles of the fifteen Spt16 mutants. Cells expressing H3-WT or H3-L61W and either wild-type or mutant Spt16, as indicated, were grown to saturation on YPD medium, harvested, resuspended in H_2_O and then spotted in a 10-fold dilution series, in which the most concentrated spots (left-most spot in each panel) contained ∼2×10^6^ cells. Plates were incubated at 30°C, except for the plate shown in the second column, which was incubated at 16°C. The approximate times of incubation were as follows: YPD 30°C, 2 days; YPD 16°C, 17 days; YPD +2% formamide (Form.), 6 days; YPD +50mM hydroxyurea (HU), 6 days; YPD +10mM caffeine (Caff.), 8 days. The strains used for these experiments are yADP1-yADP17. The results shown here are representative of two independent experiments.

As shown in Figure 2A, of the fifteen suppressor mutations we isolated, twelve harbor mutations within the Spt16-M domain, two are predicted to encode proteins with small truncations at the C-terminus and one contains a mutation in the Spt16-D domain. In addition to suppressing the Cs^−^ phenotype of H3-L61W cells, most of the Spt16 mutants also suppress several H3-L61W drug-sensitivity phenotypes (Figure 2B – note that in order to facilitate a direct comparison of the effects conferred by H3-L61W versus wild-type histone H3 (H3-WT), the strains used for these and subsequent experiments [except for those otherwise indicated] express H3-WT or H3-L61W from the *HHT2-HHF2* locus and harbor a deletion of the *HHT1-HHF1* locus, see Table S1 and Materials and Methods). The fact that many different types of mutations within the Spt16-M domain (twelve identified here plus two identified in our previous work [32]) display genetic interactions with H3-L61W strongly suggests that the Spt16-M domain is an important participant in functional interactions with histone H3. Although our screens were not saturated, the high proportion of suppressor mutations found within the Spt16-M domain compared to other regions of the protein further underscores the importance of this domain in interactions with histone H3. Interestingly, two other Spt16-M domain mutants, Spt16-E763G and Spt16-E857K, isolated based on their ability to confer dominant negative Spt^-^ phenotypes [30], were found to be unable to suppress the Cs^-^ phenotype of H3-L61W cells (data not shown), indicating that only specific mutations within the Spt16-M domain display genetic interactions with H3-L61W. The genetic interactions displayed between the Spt16-truncation mutants (Spt16-E989stop and Spt16-E1004stop) and the H3-L61W mutation (Figure 2B) also indicate a functional relationship between the extreme C-terminus of Spt16 and histone H3.

### Most of the Spt16-M domain mutants partially suppress the Spt16 3′-accumulation phenotype seen in H3-L61W cells

**Figure 3:**
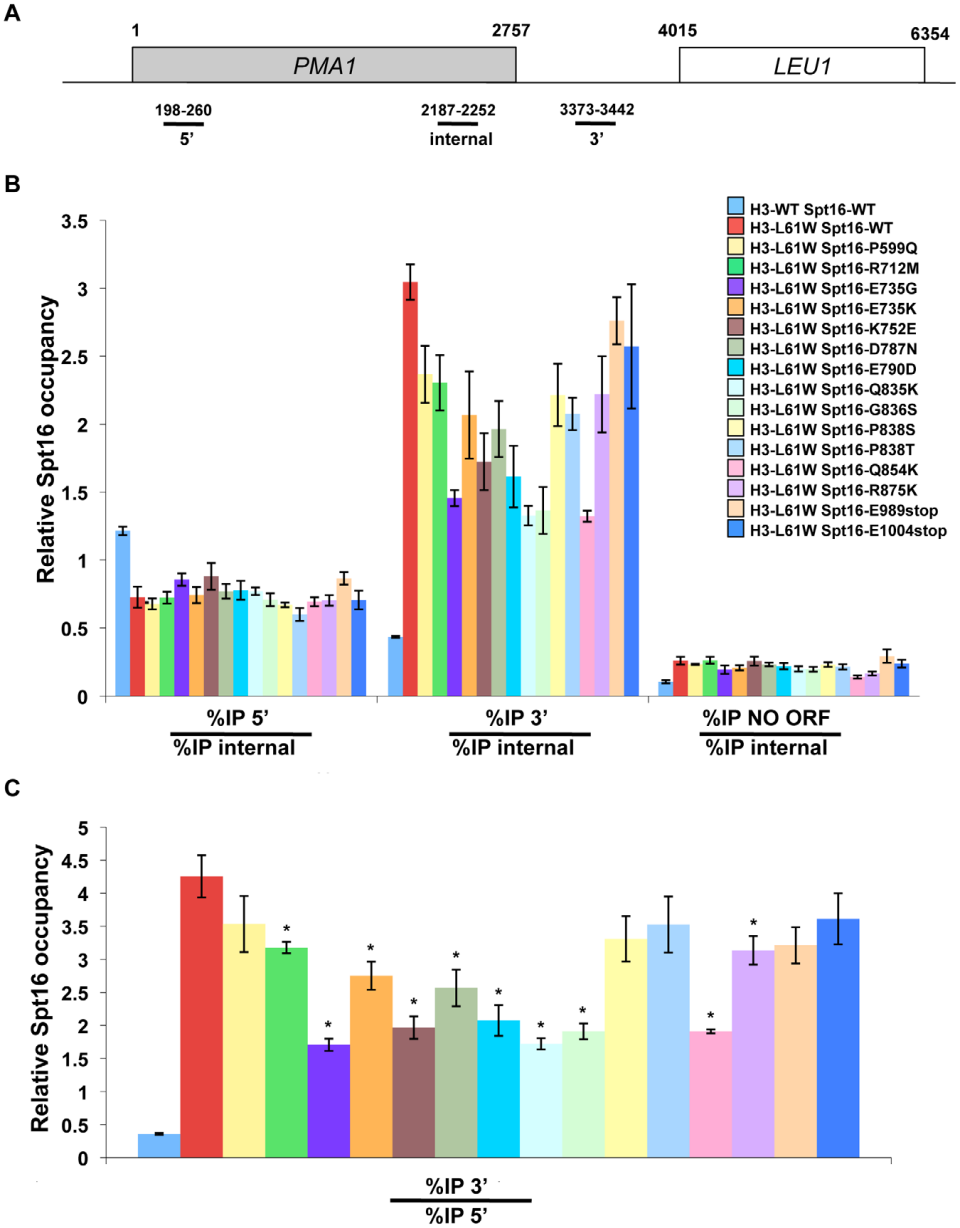
Most Spt16-M domain mutants suppress the Spt16 3′-accumulation phenotype at *PMA1* seen in H3-L61W cells. (A) Cartoon representation of the *PMA1* gene and nearby genomic regions. The shaded box represents the *PMA1* open reading frame (ORF) and the numbers above and below the diagram correspond to nucleotide positions with respect to the first nucleotide of the *PMA1* ORF, which is designated as 1. The regions analyzed in the ChIP experiments – 5′, internal, and 3′ – are indicated below the gene diagram. (B) Strains yADP1-yADP17 were subjected to ChIP experiments directed against the Spt16 protein. The % immunoprecipitation (%IP) of the three regions shown in panel A was assessed using real-time qPCR (see Materials and Methods). We previously reported that two of the standard genomic regions normally used for ChIP normalization (a non-transcribed region on chromosome V [NO ORF region] and a telomeric region on chromosome VI) are bound by Spt16 at somewhat higher levels in H3-L61W cells compared to H3-WT cells [32], [33] and are thus not ideal normalization controls for our experiments. For the studies shown here, we have instead used a region within *PMA1* itself (the “internal” region indicated in panel A) as the normalization control as we have previously shown that Spt16 occupancy levels at this location are equivalent in H3-WT and H3-L61W cells [32] and, in most cases, the Spt16 mutants bind to this region at similar levels as wild-type Spt16 in H3-WT cells (see Figure 5). For each strain, the data is shown as the mean ± S.E.M. of the ratio between the %IP of the 5′ region and the %IP of the internal region (bar-graphs on the left) and the ratio between the %IP of the 3′ region and the %IP of the internal region (bar-graphs in the middle). The binding of Spt16 to the NO ORF region is also provided for reference and, for each strain, is presented as the mean ± S.E.M. of the ratio between the %IP of the NO ORF region and the %IP of the internal region (bar-graphs on the right). (C) The data from the experiments described in panel B but expressed as a ratio between the %IP of the 3′ region and the %IP of the 5′ region for each strain. Statistically significant suppression of the Spt16 distribution defect seen in H3-L61W cells by the different Spt16 mutants was determined using a Student's *t*-test and is indicated with the asterisks (*P*<0.05).

The H3-L61W mutation causes a shift in distribution of Spt16 across transcribed genes, in which slightly lower levels of Spt16 are found at 5′ ends of genes and dramatically increased levels are seen at the 3′ ends of genes compared to what is seen in H3-WT cells [32], [33]. To obtain insights into the mechanistic nature of the suppression of H3-L61W growth phenotypes by the novel Spt16 mutants, we asked if the Spt16-distribution defect seen in H3-L61W cells is suppressed by the mutant Spt16 proteins. For these experiments, we performed chromatin immunoprecipitation (ChIP) assays to compare the levels of occupancy of Spt16 at the 5′ and 3′ ends of the *PMA1* gene in H3-WT or H3-L61W cells expressing either wild-type Spt16 or one of each of the fifteen Spt16 mutants. *PMA1* was chosen as the model gene for studying Spt16 binding patterns in our studies due to the fact that it is relatively long (thereby facilitating comparison of Spt16 binding levels at different locations across the gene), is expressed constitutively at high levels, and has been used extensively to study the transcription elongation process. As shown in Figure 3A, the 3′ region of *PMA1* assayed in our experiments overlaps with the promoter of the *LEU1* gene; however, several lines of evidence indicate that in both H3-WT and H3-L61W cells binding of Spt16 in this region is attributable to *PMA1* transcription and, at least for the most part, does not reflect events related to *LEU1* transcription. First, microarray experiments have shown that under the growth conditions used in our studies (rich medium) *LEU1* expression is minimal in H3-WT cells and this expression is unaffected by the H3-L61W mutation [31]. Second, ChIP experiments have shown that Spt16 occupancy decreases sharply in both H3-WT and H3-L61W cells downstream from the *PMA1* 3′ region [32], indicating that the Spt16 found at the *PMA1* 3′ region is not recruited to facilitate *LEU1* transcription initiation or elongation in either strain. Third, the patterns of Spt16 binding in H3-WT and H3-L61W cells at the *PMA1* 3′ region are similar to those seen at the 3′ regions of several other genes [32]. Fourth, in H3-L61W cells, Spt16 enrichment at 3′ regions of genes has been shown to be dependent on active transcription [32], consistent with the notion that high levels of Spt16 at the 3′ region of *PMA1* in H3-L61W cells is caused by *PMA1* transcription and not due to *LEU1* transcription initiation. Taken together, these observations indicate that *PMA1* serves as a good model gene with which to study the effects of H3-L61W on the distribution pattern of Spt16 across transcribed genes.

As expected, cells expressing H3-WT and wild-type Spt16 display high levels of Spt16 occupancy at the 5′ region of *PMA1* and lower occupancy levels at the 3′ region of the gene (Figure 3A and 3B). As we previously reported, Spt16 association across *PMA1* is greatly altered in the context of H3-L61W, with slightly lower levels of occupancy at the 5′ region and a marked increase in occupancy at the 3′ region when compared to H3-WT cells (Figure 3B). Interestingly, we found that the Spt16 distribution defect seen in H3-L61W cells is partially suppressed by most of the Spt16 mutants we isolated (Figure 3B). Whereas the data in Figure 3B indicate that suppression by the Spt16 mutants at *PMA1* occurs mainly by decreasing the levels of Spt16 accumulation over the 3′ region, a full appreciation of the overall level of suppression by the different Spt16 mutants is made difficult by the fact that, in some cases, suppression is also seen at the 5′ region, albeit to a much lesser degree than what is seen at the 3′ region (for example, see Spt16-E735G in Figure 3B). Therefore, to more easily visualize the full extent of suppression of the Spt16 distribution defect seen in H3-L61W by the Spt16 mutants, we expressed the ChIP data as a ratio of the levels of Spt16 binding at the *PMA1* 3′ region to the levels of Spt16 binding at the *PMA1* 5′ region. As shown in Figure 3C, ten of the twelve Spt16-M domain mutants suppress the Spt16 distribution defect seen in H3-L61W cells in a statistically significant manner. Interestingly, the Spt16-P599Q mutant and the two truncation mutants, which harbor a wild-type Spt16-M domain, do not significantly suppress the Spt16 distribution defect. Taken together, these results, combined with the fact that the two Spt16-M domain mutants we isolated in our previous work also suppress the Spt16 3′-accumulation phenotype [32], suggest that the Spt16-M domain has a role, at least in certain contexts, in controlling the dissociation of Spt16 from 3′ ends of genes following the transcription process.

### All of the Spt16 mutants isolated suppress the cryptic transcription initiation phenotype seen in H3-L61W cells

The H3-L61W mutation causes cryptic transcription initiation from within the *FLO8* gene [32], a phenotype that has been associated with defects in nucleosome reassembly during transcription elongation [34], [35], [36], [37]. To further characterize the newly isolated Spt16 mutants, we asked if any of these mutants can suppress the H3-L61W-mediated cryptic transcription initiation defect. For these experiments, we took advantage of a reporter construct that has been established to be a reliable and sensitive tool with which to assay for cryptic transcription initiation events [37]. In this reporter construct, the *HIS3* gene has been fused downstream from the cryptic promoter sequence found within the *FLO8* gene responsible for initiating cryptic transcription and the endogenous *FLO8* promoter has been replaced with the galactose-inducible *GAL1* promoter. Because the *HIS3* gene is out of frame with respect to the *FLO8* gene, growth of *his3Δ* cells in the absence of histidine is indicative of cryptic transcription [37].

**Figure 4:**
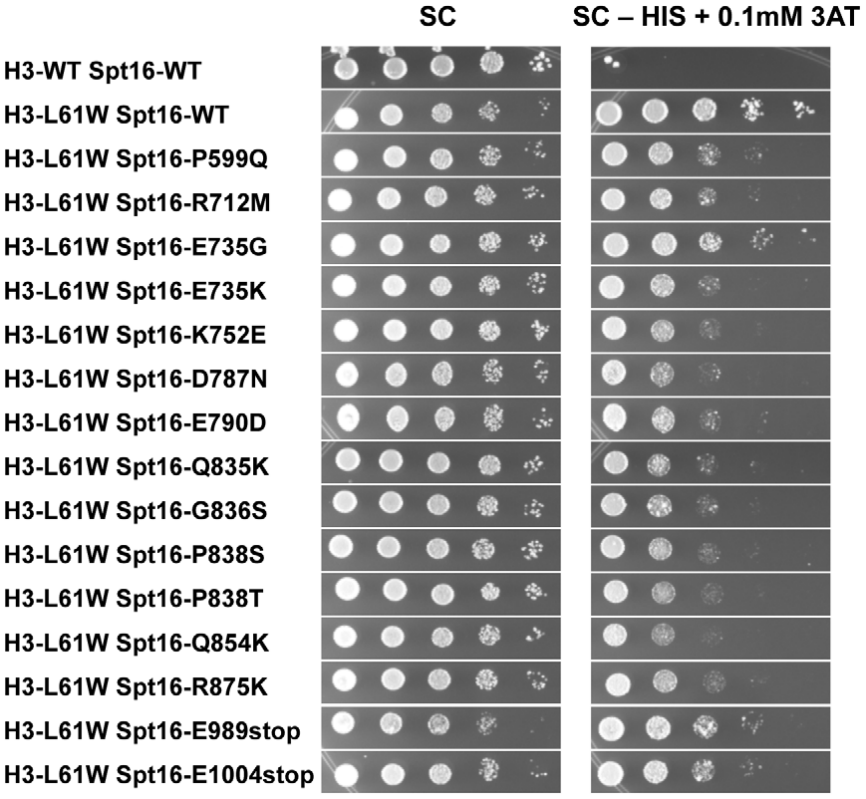
All Spt16 mutants suppress the H3-L61W-dependent cryptic transcription initiation phenotype at *FLO8*. Strains yADP18-yAPD34, all of which harbor the *FLO8-HIS3* reporter construct and express the indicated versions of the histone H3 and Spt16 proteins, were grown to saturation on YPD medium, harvested, resuspended in H_2_O and then spotted in a 10-fold dilution series, in which the most concentrated spots (left-most spot in each panel) contained ∼4×10^5^ cells. Plates were incubated at 30°C and pictures were taken after approximately 2 days of growth for the synthetic complete (SC) plate and after approximately 6 days of growth for the synthetic complete minus histidine and containing 0.1mM 3-aminotriazole, a competitive inhibitor of the *HIS3* gene product, (SC – HIS +0.1mM 3AT) plate. The results shown here are representative of three independent experiments. The suppressive effects of the Spt16 mutants were also noticeable on SC – HIS medium lacking 3AT (data not shown).

Consistent with our previous findings showing that H3-L61W causes cryptic transcription within the *FLO8* gene, H3-L61W cells harboring the *FLO8-HIS3* reporter construct displayed robust growth on medium lacking histidine compared to cells expressing H3-WT and carrying the same reporter construct (Figure 4, compare the first and second rows). When combined with any of the fifteen Spt16 mutants, H3-L61W cells showed a marked decrease in their ability to grow in the absence of histidine, despite the fact that in many cases the Spt16 mutants partially suppress the H3-L61W slow-growth phenotype seen on the SC permissive medium (Figure 4, compare second row with all the rows below it). These results indicate that the Spt16 mutants suppress the cryptic transcription initiation phenotype at *FLO8*. Interestingly, the suppression effects are only seen in conditions in which the *FLO8* gene is expected to be largely inactive (*i.e.* when cells are grown in glucose-containing medium, as in Figure 4) but not when transcription levels are expected to be high (*i.e.* when cells are grown in galactose-containing medium, data not shown), suggesting that the Spt16 mutants cannot exert their suppressive effects in the context of robust transcription.

### Characterization of the effects of the Spt16 mutants in H3-WT cells

To further elucidate the nature of our Spt16 mutants, we performed several experiments to determine their effects when expressed in cells carrying wild-type histones. In the first set of experiments, we carried out assays to ascertain the functional stability of the Spt16 mutants when exposed to different temperatures and to obtain insights into whether the mutants are defective for the process of DNA replication. For these studies, H3-WT cells expressing wild-type or mutant Spt16 proteins were assayed for growth on rich medium at 30°C, 37°C, or 16°C and on rich medium containing hydroxyurea, a drug that reduces intracellular levels of dNTPs and that is often used to score for DNA replication defects [41]. As shown in Figure S1, the Spt16 mutant cells do not show significant growth defects under these conditions, suggesting that the Spt16 mutant proteins are functionally stable at different temperatures and do not significantly impair the DNA replication process.

**Figure 5:**
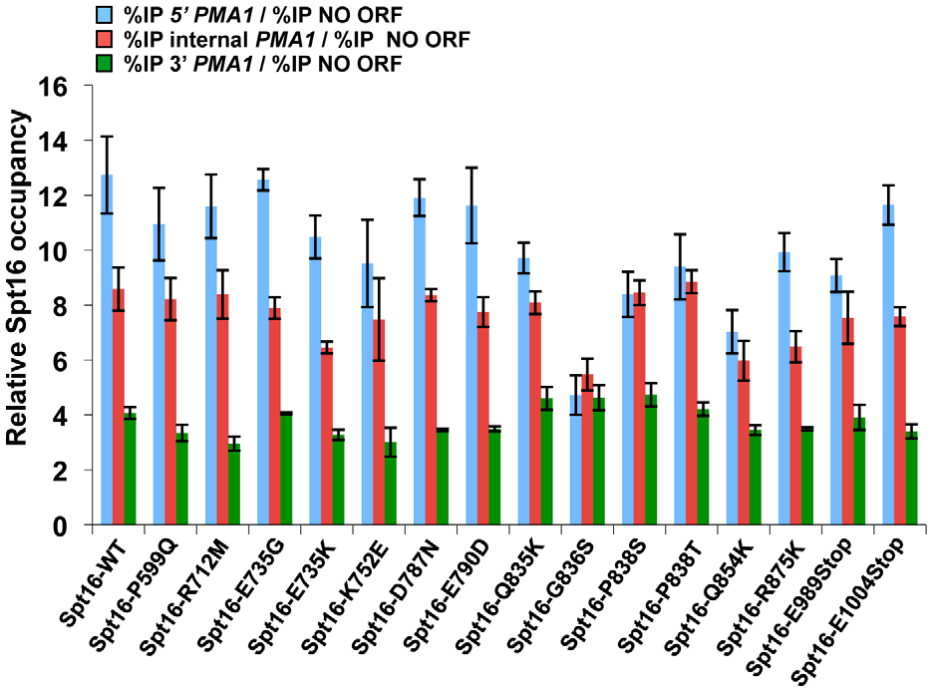
Distribution patterns of wild-type and mutant Spt16 proteins across the *PMA1* gene in H3-WT cells. ChIP experiments were carried out as described in Figure 3 on strains yAPD18 and yAPD35-yAPD49, which express H3-WT and wild-type or mutant Spt16 proteins, as indicated. For each strain, relative Spt16 occupancy at the 5′, internal and 3′ regions of *PMA1* is expressed as the mean ± S.E.M. of the ratio between the % IP of the specific *PMA1* region and the % IP of a non-transcribed region on chromosome V (NO ORF), used as a normalization control, from at least three independent experiments.

In the second set of experiments, we looked at the distribution of the mutant Spt16 proteins across the *PMA1* gene in cells expressing H3-WT. As shown in Figure 5, most of the Spt16 mutants showed a pattern of distribution similar to that seen for wild-type Spt16. However, a few mutants do appear to associate with *PMA1* in an abnormal fashion. The most defective mutant, the M-domain mutant Spt16-G836S, showed essentially the same level of occupancy at all three *PMA1* regions assayed, a pattern that is very different from that seen for wild-type Spt16 (Figure 5). These results indicate that the integrity of the Spt16-M domain is important for the ability of Spt16 to properly interact with a transcribed gene.

**Figure 6:**
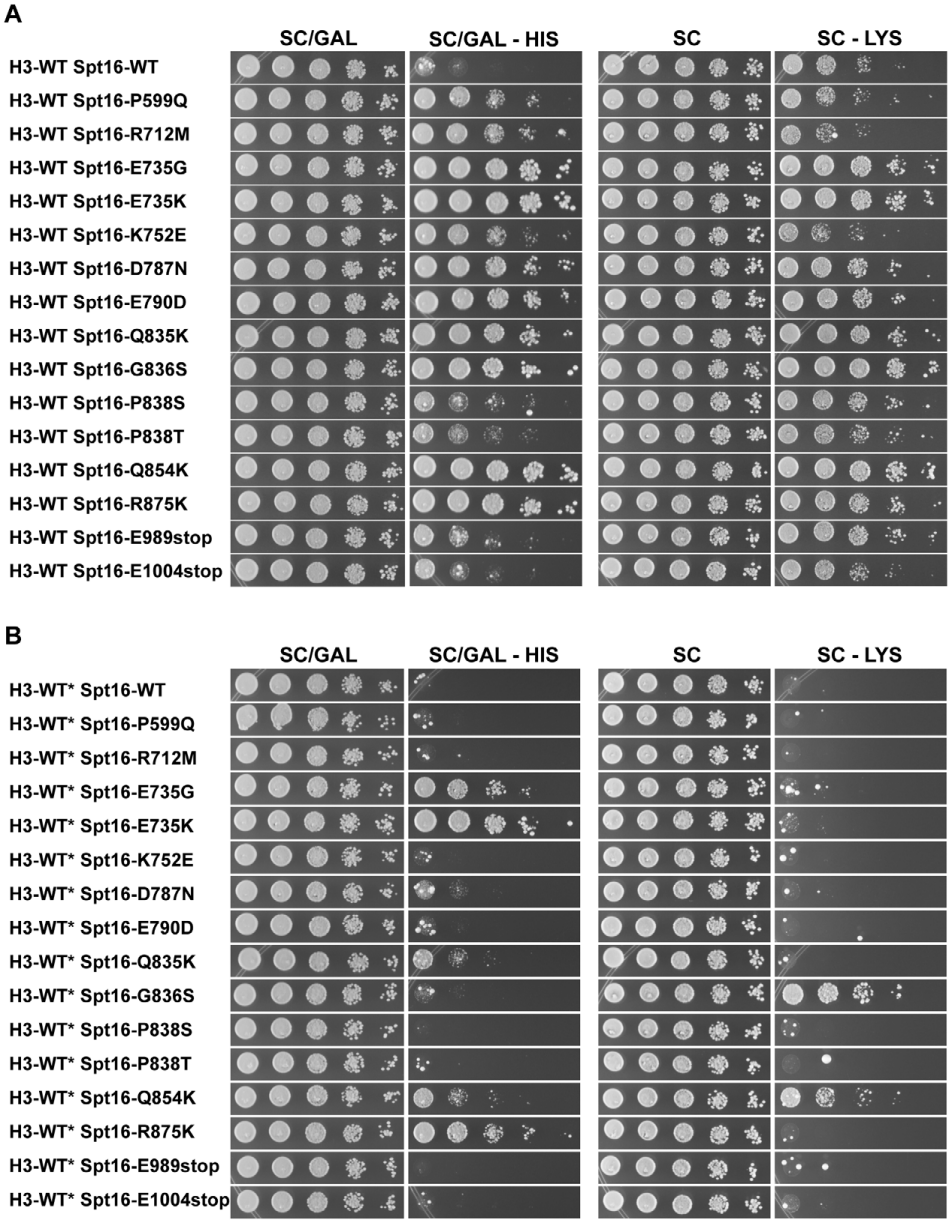
Several Spt16 mutants confer cryptic transcription initiation and Spt^-^ phenotypes in histone H3-WT cells. Strains yAPD18 and yAPD35-yAPD49, which harbor the *FLO8-HIS3* reporter construct and the *lys2-128δ* allele and express H3-WT and wild-type or mutant Spt16 proteins, as indicated, were grown to saturation on YPD medium, harvested, resuspended in H_2_O and then spotted in a 10-fold dilution series, in which the most concentrated spots (left-most spot in each panel) contained ∼4×10^5^ cells. Plates were incubated at 30°C and pictures were taken after the following approximate incubation times: synthetic complete with galactose (SC/GAL), 3 days; synthetic complete with galactose and lacking histidine (SC/GAL – HIS), 5 days; synthetic complete (SC), 2 days; synthetic complete lacking lysine (SC - LYS), 3 days. Note that the *(hht1-hhf1)Δ* mutation present in these strains' background causes a slight cryptic transcription initiation phenotype and a moderate Spt^-^ phenotype in the absence of any Spt16 mutations (see the H3-WT Spt16-WT strain). The results shown here are representative of at least three independent experiments. (B) Strains yADP51-yADP66 were subjected to the same assays as those indicated in panel (A) of this figure. The approximate incubation times were as follows: SC/GAL, 3 days; SC/GAL – HIS, 5 days; SC, 2 days; SC – LYS, 5 days. The asterisks are meant to indicate that these strains express histone H3 from both the *HHT1-HHF1* and *HHT2-HHF2* loci (see text). The results shown here are representative of at least two independent experiments

In the third set of experiments, we asked if the Spt16 mutants confer phenotypes indicative of defects in transcription and chromatin structure. For these studies, we first assessed if the Spt16 mutants cause cryptic transcription initiation defects at the *FLO8:HIS3* reporter gene in the context of H3-WT. When grown in galactose-containing medium, cells expressing one of several Spt16 mutants showed robust growth on medium lacking histidine (Figure 6A, left two panels), indicating that these mutants can cause significant levels of aberrant transcription initiation at *FLO8* in the context of H3-WT. Conversely, none of the Spt16 mutant strains grew on glucose-containing medium lacking histidine (data not shown), suggesting that the Spt16 mutants do not cause cryptic transcription initiation defects in H3-WT cells in the absence of significant levels of transcription. Next, we wished to determine if the Spt16 mutants can confer Spt^-^ phenotypes – which are associated with abnormal chromatin structure and defective transcription regulation [42] – by assaying cells carrying the *lys2-128δ* allele for growth on medium lacking lysine. As shown in the right two panels of Figure 6A, several Spt16 mutants allowed cells to grow better on this medium compared to cells expressing wild-type Spt16, indicating that these mutants confer an Spt^-^ phenotype. Interestingly, we found a general, but not perfect, correlation in the abilities of the mutants to confer the Spt^-^ and cryptic transcription initiation defects, suggesting that in some instances the two phenotypes can be related to each other. In past studies, we reported that the two original Spt16 suppressors of H3-L61W phenotypes, Spt16-E790K and Spt16-E857Q, confer an Spt^-^ phenotype only in the context of low intracellular levels of histones H3 and H4 [32]: since the experiments shown in Figure 6A were performed in the *(hht1-hhf1)Δ* background, we wished to assess if the Spt16 mutants that confer an Spt^-^ phenotype in the *(hht1-hhf1)Δ* background can also do so in the context of wild-type levels of histones H3 and H4. As shown in the right two panels of Figure 6B, only two of these mutants confer an Spt^-^ phenotype in *HHT1-HHF1* cells. Similarly, only a subset of the Spt16 mutants that cause a cryptic transcription initiation defect at the *FLO8-HIS3* reporter in *(hht1-hhf1)Δ* cells were found to also cause the cryptic transcription phenotype in cells expressing histones H3 and H4 at wild-type levels (Figure 6B, left two panels). These results indicate that several of the newly isolated Spt16 mutants can cause cryptic transcription initiation and Spt^-^ phenotypes and that, for some of these mutants, these defects are only revealed in the context of the *(hht1-hhf1)Δ* mutation, likely as a result of a more relaxed chromatin structure in this genetic background. The Spt16 mutants showing the strongest cryptic initiation defects are those harboring mutations within the Spt16-M domain, thus highlighting the importance of the integrity of this domain in preventing cryptic transcription.

### Evidence that the effects conferred by the Spt16 mutants are not attributable to changes in Spt16 abundance

**Figure 7:**
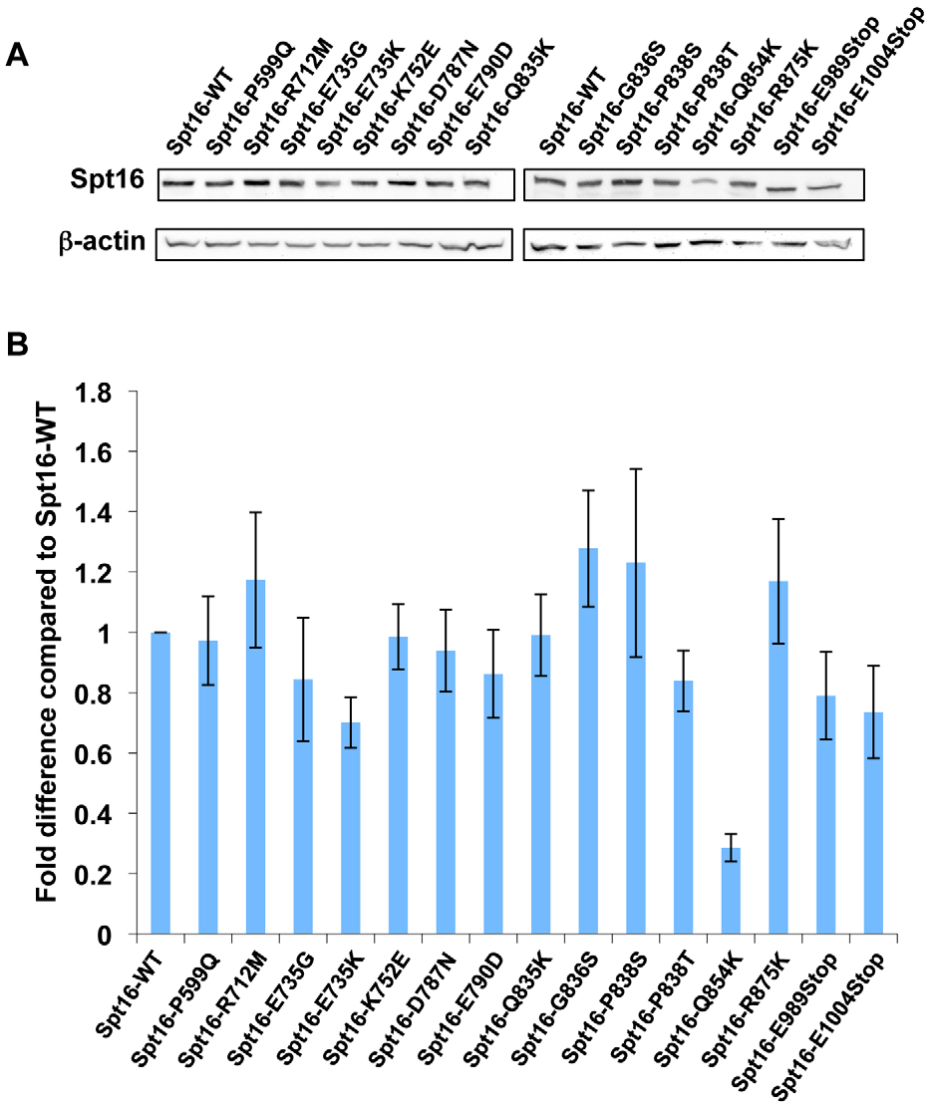
Determination of intracellular abundance of wild-type and mutant Spt16 proteins . (A) Western-blot analysis assessing the expression levels of wild-type and mutant Spt16 proteins in strains yAPD18 and yAPD35-yAPD49, which express different versions of Spt16, as indicated. Beta-actin was used as loading control for these experiments. (B) Quantitation of the abundance of wild-type and mutant Spt16 proteins. Shown are the means ± S.E.M. derived from three independent western-blot experiments. For each experiment, the level of wild-type Spt16 was arbitrarily set to 1 and, as a result, no standard error is applicable for the wild-type Spt16 samples.

To determine if the Spt16 mutants we isolated cause the various effects described in this work by affecting Spt16 protein abundance, we performed western-blot analysis to measure Spt16 protein levels in the context of the different Spt16 mutations. As shown in Figure 7, the majority of the Spt16 mutants are present in cells at levels similar to that seen for wild-type Spt16. One mutation, Q854K, does reproducibly cause a marked decrease in Spt16 abundance (Figure 7); however, whether lower protein levels are responsible for the effects seen in the context of Spt16-Q854K is unclear. The fact that most of the Spt16 mutants – including some with the strongest effects – are expressed at levels comparable to that seen for wild-type Spt16 indicates that, at least for the most part, this class of Spt16 mutants exerts its effects in a manner that is independent of alterations in Spt16 protein level.

## Discussion

In this work we have shown that the Spt16-M domain is an important player in functional interactions between Spt16 and histone H3. Mutations within the Spt16-M domain suppress several mutant growth phenotypes conferred by the histone H3 mutant H3-L61W, including growth defects at low temperatures and in the presence of several drugs. At the molecular level, many Spt16-M domain mutations suppress the Spt16 3′-accumulation phenotype at the *PMA1* gene and all Spt16-M domain mutations suppress an intragenic cryptic transcription initiation phenotype seen in H3-L61W cells. The extreme C-terminus of Spt16 is also implicated in functional interactions with histone H3 since two small C-terminal truncations of Spt16 were also found to suppress H3-L61W growth and intragenic cryptic transcription initiation phenotypes. Finally, in experiments carried out in the context of wild-type histone H3, we have provided evidence in support for roles for the Spt16-M domain in ensuring proper distribution of Spt16 across transcribed genes and in preventing aberrant cryptic transcription initiation.

The observation that the Spt16-M domain mutants suppress the Spt16 3′-accumulation phenotype at *PMA1* seen in H3-L61W cells points to a role for this domain in regulating the departure of Spt16 following transcription. However, in the context of H3-WT, the levels of Spt16 detected at the 3′ region of *PMA1* were comparable between wild-type Spt16 cells and cells expressing any of the Spt16 mutants (see green bar-graphs in Figure 5). Therefore, the Spt16 mutants do not suppress the Spt16 3′-accumulation phenotype seen in H3-L61W cells simply by an inherent and general ability of these mutants to more readily disengage from chromatin following transcription. Instead, we envision that the Spt16 suppressors may render Spt16 more sensitive to a putative Spt16-departure signal. We have previously proposed a model in which Spt16 normally requires a signal – perhaps a post-translational modification of one or more core histone residues – to be able to depart chromatin following transcription [32]. In the context of the H3-L61W mutation, this signal is either not properly initiated and/or propagated throughout the nucleosome, thereby resulting in a crippled signal that, in turn, leads to accumulation of Spt16 at 3′ ends of genes. If the Spt16-M domain mutants we have isolated are indeed more sensitive to the Spt16-departure signal, they would be expected to behave as shown in our studies: they would show more efficient departure from a gene's 3′ end in situations where the departure signal is impaired (*i.e.* in the context of H3-L61W nucleosomes), but they would have no effect in situations where the signal is fully operational (*i.e.* in the context of H3-WT nucleosomes). Thus, our results point to a role for the Spt16-M domain in regulating Spt16 dissociation from chromatin following the transcription process and are consistent with a model in which the Spt16-M domain serves as a sensor for a putative Spt16-departure signal.

Our studies show that the Spt16-M domain mutants can suppress the H3-L61W cryptic transcription initiation phenotype at the *FLO8:HIS3* reporter gene when cells are grown in glucose-containing medium while causing a cryptic transcription initiation phenotype at the same gene in the context of H3-WT when cells are grown in galactose-containing medium. These results can be explained by a model of allele-specific Spt16-histone H3 interactions. In this model, the Spt16-M domain and histone H3 interact directly to promote nucleosome reassembly during transcription elongation. The H3-L61W mutation disrupts this interaction, leading to impaired nucleosome reassembly and cryptic intragenic transcription. The Spt16-M domain mutants restore the interaction with H3-L61W through compensatory structural changes leading to more efficient nucleosome reassembly and, as a result, suppression of cryptic transcription. These structural changes, however, decrease the affinity between the Spt16-M domain and H3-WT, resulting in defective nucleosome reassembly and cryptic transcription in H3-WT cells. The Spt16-M domain may interact with histone H3 through the histone H3 N-terminal tail since this tail has been shown to interact with *S. cerevisiae* yFACT *in vitro* through yFACT regions other than the Spt16-NTD domain [26]. The observation that the Spt16 mutants show suppression of H3-L61W-dependent cryptic transcription initiation at *FLO8-HIS3* only in conditions in which the reporter gene is expected to be largely inactive seems puzzling at first given the fact that cryptic intragenic transcription is thought to be dependent on active transcription originating from a *bona fide* upstream promoter. However, recent studies have suggested that even at very lowly transcribed genes and at genes expected to be inactive, transcription-dependent nucleosome disassembly and Spt16-mediated reassembly can still occur [16]. Therefore, suppression of the H3-L61W-dependent *FLO8-HIS3* cryptic transcription phenotype by the Spt16 mutants is likely to be due to an increased ability of the Spt16 mutants to reassemble H3-L61W nucleosomes during the transcription elongation process. We do not rule out, however, alternative models that could account for cryptic transcription at *FLO8-HIS3* in H3-L61W cells grown in glucose-containing medium and for the mechanism of suppression by the Spt16 mutants. For example, it is possible that an abnormally open chromatin structure permissive to intragenic cryptic transcription is generated during DNA replication in the context of H3-L61W and that the Spt16 mutants suppress cryptic transcription by promoting a more compact chromatin structure on the newly replicated DNA molecules. Alternatively, expression of *HIS3* in the context of the *FLO8:HIS3* reporter in H3-L61W cells may be a result of changes in chromatin structure over the cryptic promoter element of *FLO8* that allows for transcription initiation to occur in the absence of any transcription originating from the *GAL1* promoter. In this scenario, the Spt16 mutants would suppress cryptic transcription by reestablishing a closed chromatin structure over the cryptic promoter element through activities related to its role in transcription initiation. Our results lay the foundations for future experiments, such as *in vitro* biochemical assays, that will further explore the significance of the interactions between the Spt16-M domain and histone H3 in regulating chromatin dynamics.

We have entertained the possibility that the abilities of Spt16 to reassemble nucleosomes during transcription elongation and to depart 3′ ends of genes following transcription are functionally related to each other. For example, it could be envisioned that in order to properly depart chromatin following transcription, Spt16 needs to efficiently reassemble the last nucleosome at the 3′ end of a transcribed gene. However, this hypothesis is not supported by our data. For example, in the context of H3-L61W nucleosomes, Spt16-E735G is among the strongest suppressors of the Spt16 3′-accumulation phenotype (Figure 3) but is one of the weakest suppressors of the cryptic transcription initiation phenotype (Figure 4). Thus, at least in this case, more efficient departure from a gene's 3′ end does not appear to correlate with a significant increase in nucleosome reassembly activity.

Our results also point to a role for the extreme C-terminus of Spt16 in directing functional interactions with histone H3. The two C-terminal truncation mutants isolated in our screens, Spt16-E989stop and Spt16-E1004stop, are predicted to lack the last 47 and 32 residues of Spt16, respectively, and western blot experiments confirm that these proteins are smaller in size compared to wild-type Spt16 (see Figure 7). The fact that these mutants are able to support life indicates that Spt16 can withstand small C-terminal truncations and still provide essential functions. In an H3-L61W background, the truncation mutants partially suppress the cryptic transcription initiation phenotype at *FLO8* (see Figure 4) but do not suppress the Spt16 3′-accumulation phenotype at *PMA1* (see Figure 3). In H3-WT cells, the truncation mutants allow for low levels of cryptic transcription initiation in the *(hht1-hhf1)Δ* background (see Figure 6A). These data suggest that the extreme C-terminal region of Spt16 is involved in the prevention of cryptic intragenic transcription and raise the possibility that the extreme C-terminus of Spt16 may influence histone deposition during transcription elongation. This region of Spt16 might exert its effects directly through interactions with histone H3 or indirectly by ensuring the structural and functional integrity of the Spt16-M domain. Alternatively, it is possible that the extreme C-terminal region of Spt16 is involved in recruitment of other factors involved in interactions with histone H3.

Our screens for Spt16 mutants able to suppress H3-L61W phenotypes were not saturated. Therefore, it is likely that mutations in residues of Spt16 other than the ones we have reported in this and our previous work can display genetic interactions with the H3-L61W mutation. An inspection of three additional Spt16 suppressors we isolated in our screens provides some relevant insights in this regard. These three mutants – Spt16-K456I, Spt16-S625L and Spt16-S841W – were found to suppress the Cs^-^ phenotypes of H3-L61W cells but were not further analyzed because each also contained a second mutation within the region targeted for the PCR mutagenesis (G3376A, C-390A and T-182Δ, respectively – see Figure 1 to locate these positions) that could potentially have an effect on *SPT16* expression levels and therefore could not be ruled out for being responsible, at least in part, for the suppression phenotypes (the only mutants with two nucleotide changes across this region that were included in the study were those in which one of the two mutations resulted in a silent mutation within the *SPT16* coding sequence, see Table 1). However, these three mutants do encode Spt16 proteins with single amino acid changes and it is therefore likely that these mutations are in fact the ones responsible for the suppression of the H3-L61W Cs^−^ phenotype. Whereas Spt16-G841W harbors a mutation within the Spt16-M domain, thereby supporting the notion that the Spt16-M domain is functionally related to histone H3, the Spt16-K456I mutation is located in a region connecting the Spt16-NTD and Spt16-D domains and the Spt16-S625L mutation is located in the Spt16-D domain. Thus, these regions may also be involved in interactions with histone H3, although the ability of Spt16-K456I or Spt16-S625L to suppress the H3-L61W-dependent Spt16 3′-accumulation phenotype and cryptic transcription initiation defect has not been assessed. That regions other than the Spt16-M domain and the extreme C-terminus might be involved in functional interactions with H3-L61W is supported by the observation that the Spt16-P599Q mutation, which is located in the Spt16-D domain, can also suppress H3-L61W phenotypes, although it is possible that this mutation and the Spt16-S625L mutation described above, due to their proximity to the Spt16-M domain, exert their effects indirectly by compromising the integrity of the Spt16-M domain. In any case, the finding that many mutations in the Spt16-M domain and two C-terminal truncations show suppression of several H3-L61W phenotypes highlights the importance of these two regions in functional interactions with histone H3. The fact that, despite the unbiased nature of the screens we carried out, a large proportion of the suppressive mutations are clustered within the Spt16-M domain further underscores the relevance of this domain in histone H3-dependent functions.

## Materials and Methods

### Yeast strains, genetic methods and media

All *S. cerevisiae* strains used in this study (Table S1) are *GAL2^+^* derivatives of the S288C strain background [43]. Most strains harbor deletions of the *HHT1-HHF1* locus (marked with either the *HIS3* or the *NatMX4* gene) and the *SPT16* gene (marked with the *KanMX4* gene) and express either H3-WT or H3-L61W from the *HHT2* or *hht2-11* alleles, respectively, located at the endogenous *HHT2* locus (see [31] for a description of the generation of the *hht2-11* strains). Strains yADP51-yADP66 express H3-WT from both the *HHT1-HHF1* and *HHT2-HHF2* loci. In addition, most strains express wild-type or mutant versions of Spt16 from a centromeric, *LEU2-*marked plasmid. The wild-type version of this plasmid (pAO01, a gift from Tim Formosa [26]) includes 400 base-pairs upstream from the *SPT16* coding sequence and 43 base-pairs of the region 3′ of the stop-codon of *SPT16* (see Figure 1). The Spt16 mutants characterized in our studies are expressed from plasmids derived from pAO01. Strain yADP50 expresses wild-type Spt16 from a centromeric, *URA3-*marked plasmid. The construction of the *KanMX4-GAL1pr-FLO8-HIS3* reporter construct (a gift from Fred Winston) has been described elsewhere [37]. Standard genetic techniques were used as described previously [44]. Details for the preparation of yeast extract peptone-dextrose (YPD), synthetic complete (SC), omission (SC-), 5-fluoroorotic acid (5-FOA), and galactose media have been described previously by others [44]. Formamide, hydroxyurea and caffeine were added to YPD medium at the concentrations indicated in the legend to Figures 2 and S1. 3-Amino-1,2,4-triazole (Sigma) was added to the SC-HIS medium presented in Figure 4 at the indicated concentration.

### Screen for Spt16 suppressors of the H3-L61W cold-sensitive phenotype

Four screens were performed to identify Spt16 mutants able to suppress the Cs^-^ phenotype conferred by the H3-L61W mutations (screens A, B, C, and D – refer to Figure 1 and Table 1). For screen A, primers OAD342 (5′ CGTAATATATTTGCATGATC) and OAD356 (5′ GTTAGCACTTTGAGAATCTTTCAG) were used to amplify the region from –773 to +1230 from plasmid pAO01. The PCR amplifications were done using *Pfu* polymerase (Stratagene) using standard PCR conditions. Two separate pools of PCR products were generated. Plasmid pAO01 was linearized with restriction enzymes KpnI and EagI (both from New England Biolabs). Strain yADP50 was co-transformed with each of the PCR pools and the linearized plasmid and cells were plated for growth on SC medium lacking leucine to select for gap-repair events generating circular plasmids. Transformants were then replica-plated to medium containing 5-FOA to select for loss of the *URA3-*marked centromeric plasmid carrying the wild-type *SPT16* gene. The resulting colonies were then replica-plated to YPD medium and incubated at 14°C to screen for suppression of the H3-L61W Cs^−^ phenotype. Candidate suppressing plasmids were then recovered from the yeast cells and retested for suppression in independent experiments.

Screens B, C, and D were carried out in a fashion similar to that described for screen A, except that the primers used were as follows: screen B, primers OAD357 (5′ GGACCTATTCTCCAATCATTCAGTCC) and OAD358 (5′ GATCCAGTCTTTTCGTTCTTCCAG), which amplify the region from +764 to +2044 (three independent pools of PCR products were obtained for this screen); screen C, primers OAD359 (5′ GAACAAAGTTACGTGGCGAAGCCCG) and OAD341 (5′ TGATTCTGTGGATAACCGTA), which amplify the region from +1430 to +3521 (three independent pools of PCR products were obtained for this screen); screen D, primers OAD342 and OAD341, which amplify the region from –773 to +3521 (four independent pools of PCR products were obtained for this screen). The enzymes used to linearize pAO01 (all from New England Biolabs) were as follows: screen B, EagI and BsaAI; screen C, SalI; screen D, KpnI and SalI.

For each screen, the estimated number of recombinant plasmids screened (see Table 1) was calculated by subtracting the number of colonies derived from transformations with linearized plasmids (and no PCR products) from the number of colonies derived from transformations in which the linearized plasmids were co-transformed with the PCR products. The region encompassing positions –773 through +3251 in each of the suppressing plasmids characterized in this study was sequenced with the assistance of the University of Arkansas for Medical Sciences (UAMS) DNA Sequencing Core Facility. In each case, both DNA strands were sequenced to ensure that the mutations identified were authentic and that they were the sole mutations present in the region.

### Chromatin immunoprecipitation (ChIP) experiments

ChIP experiments were carried out as previously described [45]. Briefly, logarithmically growing cells were cross-linked using formaldehyde (at a final concentration of 1%) and the chromatin was then collected and sheared to an average size of ∼500 base-pairs using a Misonix Sonicator 3000. Immunoprecipitations were performed overnight at 4°C using 1 µl of rabbit polyclonal antibody specific for the Spt16 protein (a gift from Tim Formosa). Antibody-Spt16-chromatin complexes were then collected using protein G sepharose beads (GE Healthcare) and washed extensively. The samples were then subjected to a step to reverse the cross-links and the precipitated DNA was then recovered and analyzed.

The % immunoprecipitation (%IP) for each of the four regions indicated in Figure 3 was determined by comparing the amount of DNA in the input samples with the amount of DNA in the samples following the Spt16-dependent immunoprecipitation step described above. Quantitation of DNA amounts was performed using a StepOnePlus Real-Time PCR system (Applied BioSystems). The primers used to amplify the regions assayed in the ChIP experiments are as follows: 5′ region of *PMA1*: OAD394 (5′ AGTTGCCGCCGGTGAA) and OAD395 (5′ CCGTAAGATGGGTCAGTTTGTAAAT); internal region of *PMA1*: OAD416 (5′ TGATGTTGCTACTTTGGCTATTGC) and OAD417 (5′ TTCCATTTAACGGGCTTTGG); 3′ region of *PMA1* OAD383 (5′ CGTAAGCGAGACTTCCAAATGG) and OAD384 (5′ TCCTGCCCAGCTCTTCTATAATACTT); NO ORF region: OAD377 (5′ TGGTGATAGGCGTTGAGTATGTG) and OAD378 (5′ GTGCGCAGTACTTGTGAAAACC). Control experiments in which the Spt16 antibody was omitted from the immunoprecipitation reactions were carried for each sample and compared with immunoprecipitation reactions in which the Spt16 antibody had been included to ensure specificity of the antibody.

### Western blot experiments

Cells were grown in liquid YPD medium to logarithmic phase and were harvested by centrifugation. Protein samples were obtained by disrupting cells using 425-600 microns acid-washed glass beads (Sigma) and strong agitation using a mini-beadbeater (Biospec Products) in protein lysis buffer (20 mM Tris-HCl, pH 7.9; 10 mM MgCl_2_, 1 mM EDTA, 5% glycerol, 0.3 M ammonium sulfate, 1 mM DTT, 1 µg /ml leupeptin, 1 µg /ml pepstatin A, and 1 mM PMSF). 50 µg (Figure 7A, left panel) or 75 µg (Figure 7A, right panel) of total protein per sample were separated by SDS-PAGE on 4-15% gradient Tris-HCl gels (Bio-Rad). Proteins were transferred onto PVDF membranes (Bio-Rad), which were then incubated in blocking solution (1X TBS, 0.1% Tween 20, and 5% non-fat dry milk). Spt16 proteins were detected by incubating blots in blocking solution containing rabbit polyclonal antibodies specific for Spt16 (a gift from Tim Formosa) at a 1∶5,000 dilution and beta-actin was detected by incubating the blots in blocking solution containing mouse monoclonal antibodies specific for beta-actin (Abcam – ab8224) at a 1∶2,000 dilution. HRP-conjugated donkey anti-rabbit secondary antibodies (Thermo Scientific – SA1-20) at a 1∶2,500 dilution were used to assay for Spt16 protein level and HRP-conjugated donkey anti-mouse rabbit antibodies (Santa Cruz Biotechnology – Sc-2096) at a 1∶2,400 dilution were used to assay for beta-actin protein levels. Detection was carried out using a SuperSignal West Pico Chemiluminescent Substrate kit (Thermo Scientific - 34080) following the manufacturer's directions and chemiluminescent signals were captured using a FluorChem FC2 system (Alpha Innotech). Quantitation of protein band intensities was carried out using the AlphaEase FC software (Alpha Innotech).

## Supporting Information

Figure S1 **Growth phenotypes of H3-WT cells expressing wild-type or mutant Spt16 proteins assayed under various conditions.** Strains yADP18, yADP19, and yADP35-49 were patched onto a YPD plate in the order shown in the table above the pictures, incubated at 30°C and then replicate plated to the plates indicated. The approximate incubation times were as follows: YPD 30°C, 1 day; YPD 37°C, 1 day; YPD 16°C, 6 days; YPD +150 mM hydroxyurea (HU), 4 days. The growth pattern of the H3-L61W Spt16-WT strain on the same plates is provided as a reference since it displays significant cold sensitivity and sensitivity to hydroxyurea.
(TIF)

Table S1 **Saccharomyces cerevisiae strains.**
(DOC)
