# Supplementary figures and images for: Mutant Versions of the S. cerevisiae Transcription Elongation Factor Spt16 Define Regions of Spt16 That Functionally Interact with Histone H3
Source: PLoS One. 2011 Jun 6;6(6):e20847. doi: 10.1371/journal.pone.0020847 (PMC3108975; doi:10.1371/journal.pone.0020847)

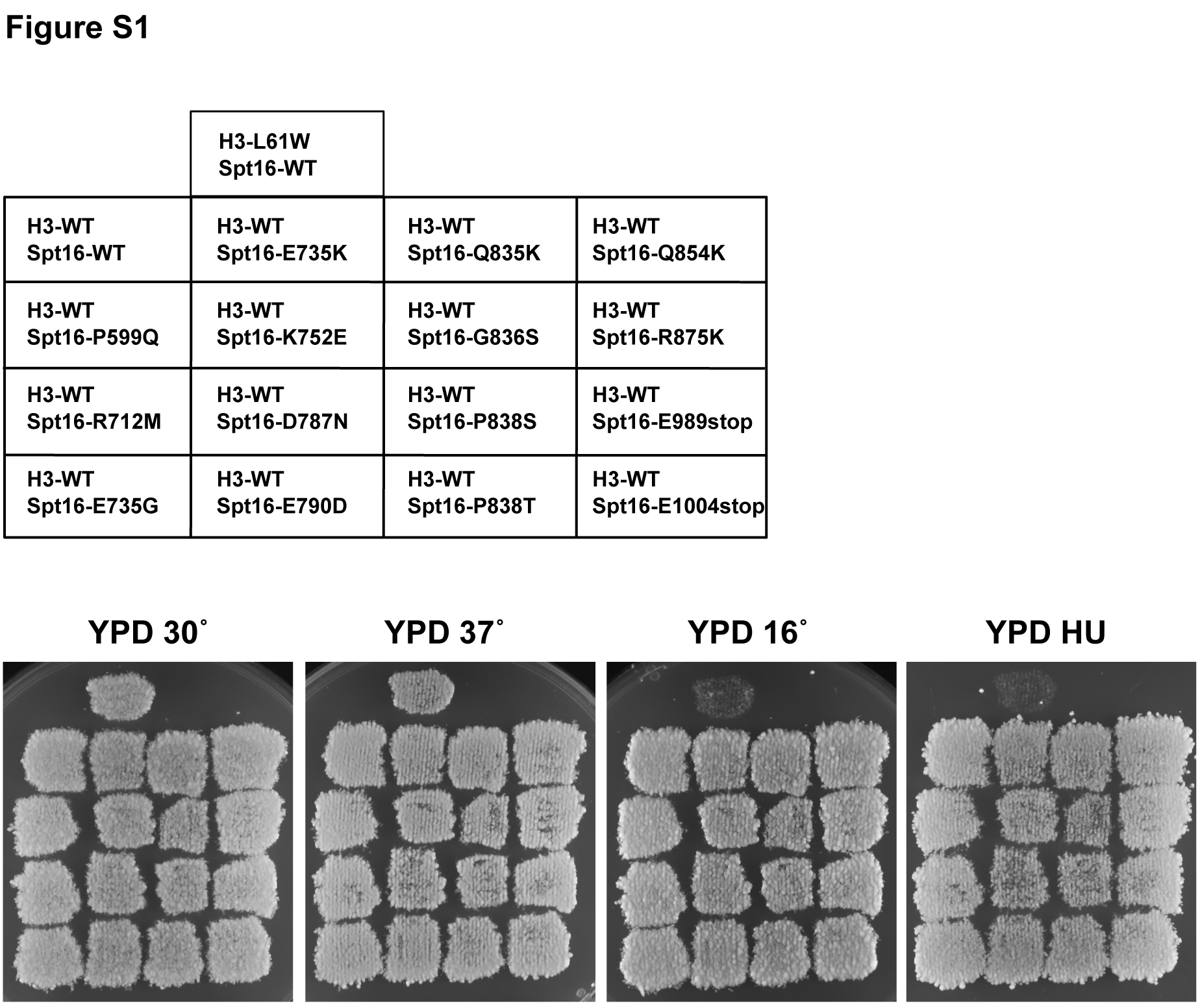

Supplement: Figure S1 — Growth phenotypes of H3-WT cells expressing wild-type or mutant Spt16 proteins assayed under various conditions. Strains yADP18, yADP19, and yADP35-49 were patched onto a YPD plate in the order shown in the table above the pictures, incubated at 30°C and then replicate plated to the plates indicated. The approximate incubation times were as follows: YPD 30°C, 1 day; YPD 37°C, 1 day; YPD 16°C, 6 days; YPD +150 mM hydroxyurea (HU), 4 days. The growth pattern of the H3-L61W Spt16-WT strain on the same plates is provided as a reference since it displays significant cold sensitivity and sensitivity to hydroxyurea. (TIF) [file pone.0020847.s001.tif]
