# Supplementary material for: Mutant Versions of the S. cerevisiae Transcription Elongation Factor Spt16 Define Regions of Spt16 That Functionally Interact with Histone H3
Source: PLoS One. 2011 Jun 6;6(6):e20847. doi: 10.1371/journal.pone.0020847 (PMC3108975; doi:10.1371/journal.pone.0020847)
Supplement: Table S1 — Saccharomyces cerevisiae strains. (DOC) [file pone.0020847.s002.doc]

Table S1. *Saccharomyces cerevisiae* strains

| Strain | Genotype |
| --- | --- |
| yADP1 | *MAT*α *his3∆200 leu2∆1 ura3-52 lys2-128δ (hht1-hhf1)∆::HIS3 HHT2 spt16∆::KanMX4 <pAO01-*(Spt16-WT*)>* |
| yADP2 | *MAT*α *his3∆200 leu2∆1 ura3-52 lys2-128δ (hht1-hhf1)∆::HIS3 hht2-11 spt16∆::KanMX4 <pAO01-*(Spt16-WT*)>* |
| yADP3 | *MAT*α *his3∆200 leu2∆1 ura3-52 lys2-128δ (hht1-hhf1)∆::HIS3 hht2-11 spt16∆::KanMX4 <pAO01-*(Spt16-P599Q*)>* |
| yADP4 | *MAT*α *his3∆200 leu2∆1 ura3-52 lys2-128δ (hht1-hhf1)∆::HIS3 hht2-11 spt16∆::KanMX4 <pAO01-*(Spt16-R712M*)>* |
| yADP5 | *MAT*α *his3∆200 leu2∆1 ura3-52 lys2-128δ (hht1-hhf1)∆::HIS3 hht2-11 spt16∆::KanMX4 <pAO01-*(Spt16-E735G*)>* |
| yADP6 | *MAT*α *his3∆200 leu2∆1 ura3-52 lys2-128δ (hht1-hhf1)∆::HIS3 hht2-11 spt16∆::KanMX4 <pAO01-*(Spt16-E735K*)>* |
| yADP7 | *MAT*α *his3∆200 leu2∆1 ura3-52 lys2-128δ (hht1-hhf1)∆::HIS3 hht2-11 spt16∆::KanMX4 <pAO01-*(Spt16-K752E*)>* |
| yADP8 | *MAT*α *his3∆200 leu2∆1 ura3-52 lys2-128δ (hht1-hhf1)∆::HIS3 hht2-11 spt16∆::KanMX4 <pAO01-(*Spt16-D787N*)>* |
| yADP9 | *MAT*α *his3∆200 leu2∆1 ura3-52 lys2-128δ (hht1-hhf1)∆::HIS3 hht2-11 spt16∆::KanMX4 <pAO01-*(Spt16-E790D*)>* |
| yADP10 | *MAT*α *his3∆200 leu2∆1 ura3-52 lys2-128δ (hht1-hhf1)∆::HIS3 hht2-11 spt16∆::KanMX4 <pAO01-*(Spt16-Q835K*)>* |
| yADP11 | *MAT*α *his3∆200 leu2∆1 ura3-52 lys2-128δ (hht1-hhf1)∆::HIS3 hht2-11 spt16∆::KanMX4 <pAO01-*(Spt16-G836S*)>* |
| yADP12 | *MAT*α *his3∆200 leu2∆1 ura3-52 lys2-128δ (hht1-hhf1)∆::HIS3 hht2-11 spt16∆::KanMX4 <pAO01-*(Spt16-P838S*)>* |
| yADP13 | *MAT*α *his3∆200 leu2∆1 ura3-52 lys2-128δ (hht1-hhf1)∆::HIS3 hht2-11 spt16∆::KanMX4 <pAO01-*(Spt16-P838T*)>* |
| yADP14 | *MAT*α *his3∆200 leu2∆1 ura3-52 lys2-128δ (hht1-hhf1)∆::HIS3 hht2-11 spt16∆::KanMX4 <pAO01-*(Spt16-Q854K*)>* |
| yADP15 | *MAT*α *his3∆200 leu2∆1 ura3-52 lys2-128δ (hht1-hhf1)∆::HIS3 hht2-11 spt16∆::KanMX4 <pAO01-*(Spt16-R875K*)>* |
| yADP16 | *MAT*α *his3∆200 leu2∆1 ura3-52 lys2-128δ (hht1-hhf1)∆::HIS3 hht2-11 spt16∆::KanMX4 <pAO01-*(Spt16-E989stop*)>* |
| yADP17 | *MAT*α *his3∆200 leu2∆1 ura3-52 lys2-128δ (hht1-hhf1)∆::HIS3 hht2-11 spt16∆::KanMX4 <pAO01-*(Spt16-E1004stop*)>* |
| yADP18 | *MAT*α  *his3∆200 leu2∆1 ura3*^1^ *lys2-128δ* *(hht1-hhf1)∆::NatMX4 HHT2 spt16∆::KanMX4 kanMX4-GAL1pr-FLO8-HIS3 <pAO01-*(Spt16-WT)*>* |
| yADP19 | *MAT*α *his3∆200 leu2∆1 ura3*^1^ *lys2-128δ* *(hht1-hhf1)∆::NatMX4 hht2-11 spt16∆::KanMX4 kanMX4-GAL1pr-FLO8-HIS3 <pAO01-*(Spt16-WT)*>* |
| yADP20 | *MAT*α *his3∆200 leu2∆1 ura3*^1^ *lys2-128δ* *(hht1-hhf1)∆::NatMX4 hht2-11 spt16∆::KanMX4 kanMX4-GAL1pr-FLO8-HIS3 <pAO01-*(Spt16*-*P599Q)*>* |
| yADP21 | *MAT*α *his3∆200 leu2∆1 ura3*^1^ *lys2-128δ* *(hht1-hhf1)∆::NatMX4 hht2-11 spt16∆::KanMX4 kanMX4-GAL1pr-FLO8-HIS3 <pAO01-*(Spt16*-*R712M)*>* |
| yADP22 | *MAT*α *his3∆200 leu2∆1 ura3*^1^ *lys2-128δ* *(hht1-hhf1)∆::NatMX4 hht2-11 spt16∆::KanMX4 kanMX4-GAL1pr-FLO8-HIS3 <pAO01-*(Spt16*-*E735G)*>* |
| yADP23 | *MAT*α *his3∆200 leu2∆1 ura3*^1^ *lys2-128δ* *(hht1-hhf1)∆::NatMX4 hht2-11 spt16∆::KanMX4 kanMX4-GAL1pr-FLO8-HIS3 <pAO01-*(Spt16*-*E735K)*>* |
| yADP24 | *MAT*α *his3∆200 leu2∆1 ura3*^1^ *lys2-128δ* *(hht1-hhf1)∆::NatMX4 hht2-11 spt16∆::KanMX4 kanMX4-GAL1pr-FLO8-HIS3 <pAO01-*(Spt16*-*K752E)*>* |
| yADP25 | *MAT*α *his3∆200 leu2∆1 ura3*^1^ *lys2-128δ* *(hht1-hhf1)∆::NatMX4 hht2-11 spt16∆::KanMX4 kanMX4-GAL1pr-FLO8-HIS3 <pAO01-*(Spt16*-*D787N)*>* |
| yADP26 | *MAT*α *his3∆200 leu2∆1 ura3*^1^ *lys2-128δ* *(hht1-hhf1)∆::NatMX4 hht2-11 spt16∆::KanMX4 kanMX4-GAL1pr-FLO8-HIS3 <pAO01-*(Spt16*-*E790D)*>* |
| yADP27 | *MAT*α *his3∆200 leu2∆1 ura3*^1^ *lys2-128δ* *(hht1-hhf1)∆::NatMX4 hht2-11 spt16∆::KanMX4 kanMX4-GAL1pr-FLO8-HIS3 <pAO01-*(Spt16*-*Q835K)*>* |
| yADP28 | *MAT*α *his3∆200 leu2∆1 ura3*^1^ *lys2-128δ* *(hht1-hhf1)∆::NatMX4 hht2-11 spt16∆::KanMX4 kanMX4-GAL1pr-FLO8-HIS3 <pAO01-*(Spt16*-*G836S)*>* |
| yADP29 | *MAT*α *his3∆200 leu2∆1 ura3*^1^ *lys2-128δ* *(hht1-hhf1)∆::NatMX4 hht2-11 spt16∆::KanMX4 kanMX4-GAL1pr-FLO8-HIS3 <pAO01-*(Spt16*-*P838S)*>* |
| yADP30 | *MAT*α *his3∆200 leu2∆1 ura3*^1^ *lys2-128δ* *(hht1-hhf1)∆::NatMX4 hht2-11 spt16∆::KanMX4 kanMX4-GAL1pr-FLO8-HIS3 <pAO01-*(Spt16*-*P838T)*>* |
| yADP31 | *MAT*α *his3∆200 leu2∆1 ura3*^1^ *lys2-128δ* *(hht1-hhf1)∆::NatMX4 hht2-11 spt16∆::KanMX4 kanMX4-GAL1pr-FLO8-HIS3 <pAO01-*(Spt16*-*Q854K)*>* |
| yADP32 | *MAT*α *his3∆200 leu2∆1 ura3*^1^ *lys2-128δ* *(hht1-hhf1)∆::NatMX4 hht2-11 spt16∆::KanMX4 kanMX4-GAL1pr-FLO8-HIS3 <pAO01-*(Spt16*-*R875K)*>* |
| yADP33 | *MAT*α *his3∆200 leu2∆1 ura3*^1^ *lys2-128δ* *(hht1-hhf1)∆::NatMX4 hht2-11 spt16∆::KanMX4 kanMX4-GAL1pr-FLO8-HIS3 <pAO01-*(Spt16*-*E989stop)*>* |
| yADP34 | *MAT*α *his3∆200 leu2∆1 ura3*^1^ *lys2-128δ* *(hht1-hhf1)∆::NatMX4 hht2-11 spt16∆::KanMX4 kanMX4-GAL1pr-FLO8-HIS3 <pAO01-*(Spt16*-*E1004stop)*>* |
| yADP35 | *MAT*α *his3∆200 leu2∆1 ura3*^1^ *lys2-128δ* *(hht1-hhf1)∆::NatMX4 HHT2 spt16∆::KanMX4 kanMX4-GAL1pr-FLO8-HIS3 <pAO01-*(Spt16-P599Q)*>* |
| yADP36 | *MAT*α *his3∆200 leu2∆1 ura3*^1^ *lys2-128δ* *(hht1-hhf1)∆::NatMX4 HHT2 spt16∆::KanMX4 kanMX4-GAL1pr-FLO8-HIS3 <pAO01-*(Spt16-R712M)*>* |
| yADP37 | *MAT*α *his3∆200 leu2∆1 ura3*^1^ *lys2-128δ* *(hht1-hhf1)∆::NatMX4 HHT2 spt16∆::KanMX4 kanMX4-GAL1pr-FLO8-HIS3 <pAO01-*(Spt16-E735G)*>* |
| yADP38 | *MAT*α *his3∆200 leu2∆1 ura3*^1^ *lys2-128δ* *(hht1-hhf1)∆::NatMX4 HHT2 spt16∆::KanMX4 kanMX4-GAL1pr-FLO8-HIS3 <pAO01-*(Spt16-E735K)*>* |
| yADP39 | *MAT*α *his3∆200 leu2∆1 ura3*^1^ *lys2-128δ* *(hht1-hhf1)∆::NatMX4 HHT2 spt16∆::KanMX4 kanMX4-GAL1pr-FLO8-HIS3 <pAO01-*(Spt16-K752E)*>* |
| yADP40 | *MAT*α *his3∆200 leu2∆1 ura3*^1^ *lys2-128δ* *(hht1-hhf1)∆::NatMX4 HHT2 spt16∆::KanMX4 kanMX4-GAL1pr-FLO8-HIS3 <pAO01-*(Spt16-D787N)*>* |
| yADP41 | *MAT*α *his3∆200 leu2∆1 ura3*^1^ *lys2-128δ* *(hht1-hhf1)∆::NatMX4 HHT2 spt16∆::KanMX4 kanMX4-GAL1pr-FLO8-HIS3 <pAO01-*(Spt16-E790D)*>* |
| yADP42 | *MAT*α *his3∆200 leu2∆1 ura3*^1^ *lys2-128δ* *(hht1-hhf1)∆::NatMX4 HHT2 spt16∆::KanMX4 kanMX4-GAL1pr-FLO8-HIS3 <pAO01-*(Spt16-Q835K)*>* |
| yADP43 | *MAT*α *his3∆200 leu2∆1 ura3*^1^ *lys2-128δ* *(hht1-hhf1)∆::NatMX4 HHT2 spt16∆::KanMX4 kanMX4-GAL1pr-FLO8-HIS3 <pAO01-*(Spt16-G836S)*>* |
| yADP44 | *MAT*α *his3∆200 leu2∆1 ura3*^1^ *lys2-128δ* *(hht1-hhf1)∆::NatMX4 HHT2 spt16∆::KanMX4 kanMX4-GAL1pr-FLO8-HIS3 <pAO01-*(Spt16-P838S)*>* |
| yADP45 | *MAT*α *his3∆200 leu2∆1 ura3*^1^ *lys2-128δ* *(hht1-hhf1)∆::NatMX4 HHT2 spt16∆::KanMX4 kanMX4-GAL1pr-FLO8-HIS3 <pAO01-*(Spt16-P838T)*>* |
| yADP46 | *MAT*α *his3∆200 leu2∆1 ura3*^1^ *lys2-128δ* *(hht1-hhf1)∆::NatMX4 HHT2 spt16∆::KanMX4 kanMX4-GAL1pr-FLO8-HIS3 <pAO01-*(Spt16-Q854K)*>* |
| yADP47 | *MAT*α *his3∆200 leu2∆1 ura3*^1^ *lys2-128δ* *(hht1-hhf1)∆::NatMX4 HHT2 spt16∆::KanMX4 kanMX4-GAL1pr-FLO8-HIS3 <pAO01-*(Spt16-R875K)*>* |
| yADP48 | *MAT*α *his3∆200 leu2∆1 ura3*^1^ *lys2-128δ* *(hht1-hhf1)∆::NatMX4 HHT2 spt16∆::KanMX4 kanMX4-GAL1pr-FLO8-HIS3 <pAO01-*(Spt16-E989stop)*>* |
| yADP49 | *MAT*α *his3∆200 leu2∆1 ura3*^1^ *lys2-128δ* *(hht1-hhf1)∆::NatMX4 HHT2 spt16∆::KanMX4 kanMX4-GAL1pr-FLO8-HIS3 <pAO01-*(Spt16-E1004stop)*>* |
| yADP50 | *MAT*α *his3∆200 leu2∆1 ura3-52 lys2-128δ (hht1-hhf1)∆::HIS3 hht2-11 spt16∆::KanMX4 <pSPT16-URA3>* |
| yADP51 | *MAT*α *his3∆200 leu2∆1 ura3*^1^ *lys2-128δ* *HHT2 spt16∆::KanMX4 kanMX4-GAL1pr-FLO8-HIS3 <pAO01-*(Spt16-WT)*>* |
| yADP52 | *MAT*α *his3∆200 leu2∆1 ura3*^1^ *lys2-128δ* *HHT2 spt16∆::KanMX4 kanMX4-GAL1pr-FLO8-HIS3 <pAO01-*(Spt16-P599Q)*>* |
| yADP53 | *MAT*α *his3∆200 leu2∆1 ura3*^1^ *lys2-128δ* *HHT2 spt16∆::KanMX4 kanMX4-GAL1pr-FLO8-HIS3 <pAO01-*(Spt16-R712M)*>* |
| yADP54 | *MAT*α *his3∆200 leu2∆1 ura3*^1^ *lys2-128δ* *HHT2 spt16∆::KanMX4 kanMX4-GAL1pr-FLO8-HIS3 <pAO01-*(Spt16-E735G)*>* |
| yADP54 | *MAT*α *his3∆200 leu2∆1 ura3*^1^ *lys2-128δ* *HHT2 spt16∆::KanMX4 kanMX4-GAL1pr-FLO8-HIS3 <pAO01-*(Spt16-E735K)*>* |
| yADP56 | *MAT*α *his3∆200 leu2∆1 ura3*^1^ *lys2-128δ* *HHT2 spt16∆::KanMX4 kanMX4-GAL1pr-FLO8-HIS3 <pAO01-*(Spt16-K752E)*>* |
| yADP57 | *MAT*α *his3∆200 leu2∆1 ura3*^1^ *lys2-128δ* *HHT2 spt16∆::KanMX4 kanMX4-GAL1pr-FLO8-HIS3 <pAO01-*(Spt16-D787N)*>* |
| yADP58 | *MAT*α *his3∆200 leu2∆1 ura3*^1^ *lys2-128δ* *HHT2 spt16∆::KanMX4 kanMX4-GAL1pr-FLO8-HIS3 <pAO01-*(Spt16-E790D)*>* |
| yADP59 | *MAT*α *his3∆200 leu2∆1 ura3*^1^ *lys2-128δ* *HHT2 spt16∆::KanMX4 kanMX4-GAL1pr-FLO8-HIS3 <pAO01-*(Spt16-Q835K)*>* |
| yADP60 | *MAT*α *his3∆200 leu2∆1 ura3*^1^ *lys2-128δ* *HHT2 spt16∆::KanMX4 kanMX4-GAL1pr-FLO8-HIS3 <pAO01-*(Spt16-G836S)*>* |
| yADP61 | *MAT*α *his3∆200 leu2∆1 ura3*^1^ *lys2-128δ* *HHT2 spt16∆::KanMX4 kanMX4-GAL1pr-FLO8-HIS3 <pAO01-*(Spt16-P838S)*>* |
| yADP62 | *MAT*α *his3∆200 leu2∆1 ura3*^1^ *lys2-128δ* *HHT2 spt16∆::KanMX4 kanMX4-GAL1pr-FLO8-HIS3 <pAO01-*(Spt16-P838T)*>* |
| yADP63 | *MAT*α *his3∆200 leu2∆1 ura3*^1^ *lys2-128δ* *HHT2 spt16∆::KanMX4 kanMX4-GAL1pr-FLO8-HIS3 <pAO01-*(Spt16-Q854K)*>* |
| yADP64 | *MAT*α *his3∆200 leu2∆1 ura3*^1^ *lys2-128δ* *HHT2 spt16∆::KanMX4 kanMX4-GAL1pr-FLO8-HIS3 <pAO01-*(Spt16-R875K)*>* |
| yADP65 | *MAT*α *his3∆200 leu2∆1 ura3*^1^ *lys2-128δ* *HHT2 spt16∆::KanMX4 kanMX4-GAL1pr-FLO8-HIS3 <pAO01-*(Spt16-E989stop)*>* |
| yADP66 | *MAT*α *his3∆200 leu2∆1 ura3*^1^ *lys2-128δ* *HHT2 spt16∆::KanMX4 kanMX4-GAL1pr-FLO8-HIS3 <pAO01-*(Spt16-E1004stop)*>* |

^1^The allele at this locus is either *ura3-52* or *ura3∆0.*
